# Food-dependent exercise-induced anaphylaxis to chickpea in a 17-year-old female: a case report

**DOI:** 10.1186/s13256-015-0669-6

**Published:** 2015-09-03

**Authors:** Hannah Roberts, Moshe Ben-Shoshan

**Affiliations:** 1 Department of Pediatrics, Janeway Children’s Hospital, 300 Prince Phillip Drive, St. John’s, Newfoundland and Labrador A1B 3V6 Canada; 2 Division of Allergy and Clinical Immunology, Department of Pediatrics, Montreal Children’s Hospital, Montreal, Quebec Canada

## Abstract

**Introduction:**

Food-dependent exercise-induced anaphylaxis is a subtype of anaphylaxis and, although rare, it is an important condition to be familiar with as it can ultimately lead to death.

**Case presentation:**

We present a case of food-dependent exercise-induced anaphylaxis in a 17-year-old white girl due to chickpea. She had a history of anaphylaxis after eating crackers and hummus before exercising. Skin prick testing and serum-specific immunoglobulin E level confirmed chickpea to be the causative allergen.

**Conclusions:**

This case demonstrates the challenge in identifying specific causative food allergens when foods are eaten in combination, when the food is processed, and when cross-reactivity is possible. These challenges add complexity to a condition that is already rare and unfamiliar to some health care providers. We hope that this case will serve as an important reminder that although rare, food-dependent exercise-induced anaphylaxis exists and making a diagnosis can lead to life-saving preventative strategies. As legumes are not a common food associated with food-dependent exercise-induced anaphylaxis, this will add to our current knowledge base in the field of allergy.

## Introduction

Anaphylaxis is a systemic allergic reaction that is rapid in onset and has the potential to cause death [1]. Once diagnosed, avoidance of allergen and carrying an epinephrine auto-injector is recommended [2]. Most anaphylactic reactions are immunoglobulin E (IgE) mediated and the major triggers include food, medication, venom, latex, exercise, and transfusions [3]. It is reported that anaphylaxis affects at least 1.6% of the general population [4].

Food-dependent exercise-induced anaphylaxis (FDEIA) is a subtype of anaphylaxis and is rare [5]. FDEIA is more commonly described in adolescents and adults versus younger children [6]. The condition is characterized by anaphylaxis that develops in association with physical exertion and ingestion of a causative food within a certain timeframe. In an analysis of 167 Japanese cases of FDEIA, 80% of the patients developed symptoms within 2 hours of eating the causative food [7]. Neither the food allergen nor exercise alone triggers anaphylaxis. Typical symptoms seen in FDEIA include skin manifestations (urticaria, erythema, edema, and pruritus), dyspnea, abdominal pain, and fatigue [8]. The pathogenesis is not fully understood yet. Based on skin prick testing (SPT) and specific IgE results for causative foods, an IgE mechanism is likely. The exact mechanism that results in a transient disruption in immune tolerance to causative foods is not known and different theories exist [9]. It is thought that exertion triggers physiological change that enhances absorption of undigested, immunoreactive forms of allergen from the gastrointestinal tract. Specific co-triggers such as non-steroidal anti-inflammatory drugs (NSAIDS), aspirin, extreme temperatures, a second food, menstruation, and stress, have also been theorized to aid in the development of FDEIA [5, 6]. The primary foods reported to trigger FDEIA are wheat and shellfish [10], although in Europe tomatoes appear to be more common in FDEIA than wheat [6]. A variety of other foods have been identified in FDEIA including vegetables, fruits, nuts, egg, mushrooms, rice, and meat [7].

Diagnosis relies on a thorough history to identify food allergen exposure, along with the combination of exercise and possible co-triggers. SPT and specific IgE levels can reveal the food allergen(s) and exclude other suspected allergens. A positive oral-food exercise challenge would further confirm a diagnosis, but is unnecessary if the history is suggestive and SPT and/or IgE levels are consistent [5].

We present a case of FDEIA to chickpea in a 17-year-old girl with a convincing clinical history, positive SPT to fresh chickpea and hummus extract, along with an elevated serum-specific IgE level to chickpea. To the best of our knowledge, this is the first case demonstrating FDEIA to chickpea in an adolescent. This case describes the challenge in identifying specific causative food allergens when foods are eaten in combination, when the food is processed, and when cross-reactivity is possible.

## Case presentation

A 17-year-old white girl was taken to the emergency room by ambulance when she developed anaphylaxis at home following exercise. In terms of potential food allergens, she ate five rice crackers containing rice, wheat, and soy oil, along with hummus containing chickpeas and sesame. Approximately 10 minutes after eating the crackers and hummus she ran on a treadmill. Within 10 minutes from onset of physical activity, she developed lip swelling and stopped running. She proceeded to develop periorbital edema, urticaria, generalized pruritus, and abdominal pain. In the emergency department, her vital signs were within normal range. She was treated with Benadryl (diphenhydramine) and intramuscular epinephrine approximately 1 hour after ingestion of the food. Symptoms resolved within approximately 3 hours.

She consumed the specific crackers on one previous occasion, not associated with exercise, without any reaction. On multiple occasions she has tolerated hummus; however, details regarding previous combination of hummus and exercise are unknown. On numerous occasions she has tolerated rice, wheat, soybean, chickpea and sesame. She regularly exercises and has not had issues with postprandial activity. There was no past personal history of anaphylaxis, angioedema, food allergy, atopic dermatitis, drug allergy, or vaccine allergy. She had been well that day with no concurrent illness. She was not exposed to other foods, alcohol, or medication (including NSAIDs, aspirin) several hours prior to exercise. She was not menstruating, and there was no exposure to extreme temperature changes.

### Investigations

**Fig. 1:**
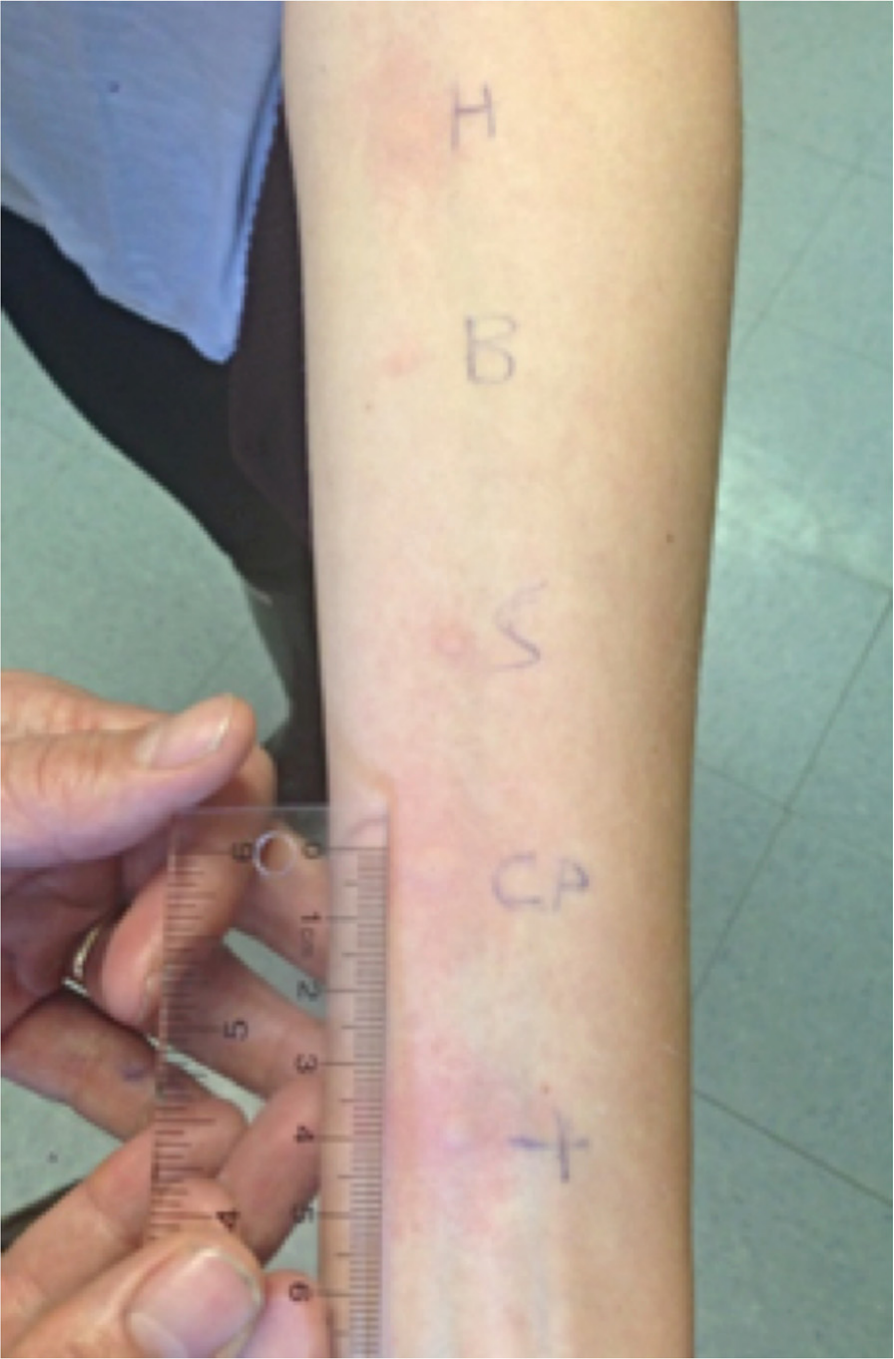
Results of skin prick testing. From top, hummus (*H*) 7×15mm (wheal/erythema), cracker (*B*) negative, soy (*S*) 4×6mm, chickpea (*CP*) 7×15mm, and lastly histamine positive control (*+*) 5×10mm

In the out-patient allergy clinic, SPT was performed for ingredients in the meal consumed prior to exercise including wheat, rice, soybean, sesame, and fresh chickpea, as well as for the specific hummus and crackers ingested prior to the reaction. SPT was positive for soybean, chickpeas, and hummus, but negative for the other suspected allergens (Fig. 1). Specific IgE levels for soybean and chickpea were 6.32kU/L and 4.14kU/L respectively. Serum level for tryptase at baseline was within normal limits (4.1mcg/L, normal range: 0.0 to 13.5mcg/L).

### Differential diagnosis

The patient presented with cutaneous and gastrointestinal symptoms within minutes of exposure to suspected allergen that was tolerated in the absence of exercise and hence meets the definition of FDEIA. Given that she has never developed symptoms of anaphylaxis in the context of exercise or after eating the culprit food independently prior to this episode as well as afterwards, this is unlikely to be exercise-induced anaphylaxis or sole food allergy. Given that her symptoms were not limited to her skin, cholinergic urticaria is an unlikely diagnosis in this case. She did not have any other concerning exposures such as venom or temperature extremes and she did not take any NSAIDs or aspirin that could have elicited the reaction. The negative SPT to the cracker extract narrows the differential for the causative food, which further suggested that chickpea in combination with exercise caused the incident. Together, this supports FDEIA as the most probable diagnosis.

### Treatment

The patient and her mother were educated about the potential consequences of FDEIA. It was recommended that she avoid eating chickpeas or anything containing chickpea such as hummus 2 hours before and up to 2 hours after exercising. She will carry an epinephrine auto-injector and was educated on proper use of the device. It was recommended that her family and her school be made aware of her condition, and an emergency action plan was reviewed.

### Outcome and follow-up

Since the initial visit, the patient has not experienced anaphylaxis. The exact crackers and hummus, as well as fresh chickpeas, have been consumed and tolerated without exercise. Exercise has been well tolerated. She will be followed-up again 3 months after her initial visit.

## Discussion

To the best of our knowledge, this is the first case demonstrating FDEIA due to chickpea in an adolescent patient, and only the second case overall reporting FDEIA due to chickpea. The first case was identified in a 41-year-old woman after dancing [11]. Legumes are not a common food associated with FDEIA, and there are minimal reports in the literature. Of interest, Orhan and Karakas discuss a 17-year-old with FDEIA to lentils [9]. Adachi *et al.* describe the first known case of FDEIA induced by soybean products in a 16-year-old girl who ate tofu [8]. So far, FDEIA is more common in adolescents and adults, but should not be overlooked when assessing younger patients [6].

This case demonstrates the challenge in identifying specific causative food allergens when foods are eaten in combination and when a potential allergen is contained in processed food. Rice crackers and hummus were the suspected foods and therefore several potential allergens were investigated based on their ingredients: wheat, rice, soy oil, sesame, and chickpeas. There was no previous history of food allergy to any of these food items and SPT, given its high sensitivity, excluded wheat, rice, and sesame allergy [12]. The extracts prepared from the actual food products were helpful in excluding the cracker, which contained soy oil. It is reported that food processing may affect the risk of reaction to the food allergen. Adachi *et al.* demonstrated the variation in allergen exposure depending on the form of food [8]. They report a case of FDEIA due to tofu in a patient who tolerated soymilk and identified β-conglycinin as the specific allergen. Immunoblot analysis demonstrated β-conglycinin in soymilk completely disappeared after pepsin digestion within 20 minutes, whereas β-conglycinin in tofu was almost intact after more than 120 minutes of pepsin digestion [8]. We have not identified which allergenic protein(s) in chickpea was responsible for the reaction in our case and we do not know if chickpea found in other foods would produce the same effect. Therefore, our recommendations were to avoid chickpeas and any chickpea-containing products in combination with exercise.

The cracker contained soy oil and initially the question of whether soy oil was capable of triggering FDEIA was considered. SPT to the cracker extract was negative, suggesting this was unlikely in our case. Processed soybean oil is typically considered safe for patients with soy allergy. Soy oil is usually produced from the hexane extract of soybean and some of the soybean proteins are included in the extract, therefore the potential for allergy exists. Awazuhara *et al.* investigated the IgE and immunoglobulin G4 (IgG4)-binding abilities of soy oil proteins and reported that proteins in soy oil have little antigenicity with respect to soybean allergy [13]. The risk of residual protein in soybean oil triggering an allergic reaction in soy allergic individuals has not been extensively studied, and the question remains of whether exercise or other triggers could have an effect on the antigenicity of residual proteins in soy oil.

When legumes are eaten in combination, the potential for cross-reactivity also contributes complexity to identifying the causative allergen(s). Our patient had both positive skin prick tests and elevated serum-specific IgE levels to chickpea and soybean. Testing using extracts from the suspected food was helpful in confirming chickpea as the causal food. It is common to have positive IgE antibody tests to several legumes in those who only clinically react to one type of legume. Oral food challenges have been used to assess clinical allergy to legumes and in the past they have demonstrated that clinical cross-reactivity to legumes in children is rare [14]. However, recent studies suggest serological and clinical legume (that is, lentils, chickpeas, and peas) cross-reactivity is common [15]. With so few cases of FDEIA due to legume, further studies are warranted to look at legume cross-reactivity specifically in FDEIA.

## Conclusions

To the best of our knowledge, this is the first case demonstrating FDEIA to chickpea in an adolescent patient. This case demonstrates the challenge in identifying specific causative food allergens when foods are eaten in combination, when the food is processed, and when cross-reactivity is possible. These challenges add complexity to a condition, FDEIA, which is already rare and unfamiliar to some health care providers. We hope that this case will serve as an important reminder that, although rare, FDEIA exists and making a diagnosis can lead to life-saving preventative strategies. As legumes are not a common food associated with FDEIA, this will add to our current knowledge base in the field of allergy.

## Consent

Written informed consent was obtained from the patient’s legal guardian(s) for the publication of this case report and any accompanying images. A copy of the written consent is available for review by the Editor-in-Chief of this journal.

## Abbreviations

FDEIA: Food-dependent exercise-induced anaphylaxis
IgE: Immunoglobulin E
NSAID: Non-steroidal anti-inflammatory drug
SPT: Skin prick testing
